# Unmet Need for Family Planning among Urban and Rural Married Women in Yangon Region, Myanmar—a Cross-Sectional Study

**DOI:** 10.3390/ijerph16193742

**Published:** 2019-10-04

**Authors:** Myint Myint Wai, Espen Bjertness, Hein Stigum, Thein Thein Htay, Tippawan Liabsuetrakul, Aye Nyein Moe Myint, Johanne Sundby

**Affiliations:** 1 Ministry of Health and Sports, NayPyiTaw 15011, Myanmar; htaythein90@gmail.com (T.T.H.); anmoemyint@gmail.com (A.N.M.M.); 2 Department of Community Medicine and Global Health, University of Oslo, 0318 Oslo, Norway; espen.bjertness@medisin.uio.no (E.B.); hein.stigum@medisin.uio.no (H.S.); johanne.sundby@medisin.uio.no (J.S.); 3 Epidemiology Unit, Faculty of Medicine, Prince of Songkla University, Hat Yai, Songkhla 90110, Thailand; ltippawa@yahoo.com

**Keywords:** contraceptives, unmet need for family planning, demand, satisfaction, urban and rural women

## Abstract

Despite increasing contraceptive use and prevalence, many women who want to avoid or delay pregnancy are not using contraceptives. This results in unintended pregnancies, which increases the risk of unsafe abortions. This study aimed to explore the extent of the unmet need for family planning (FP) among urban and rural married women in Myanmar and their demand for and satisfaction with FP. A cross-sectional survey using adapted Demographic and Health surveys questions was conducted in south and north Yangon from September 2016 to November 2016. A total of 1100 currently married women of 18–49 years participated. The contraceptive prevalence was 67.2% in total, 63% urban, and 70% rural. About 19.4% (95% CI: 16.7%–22.4%) of the studied women had an unmet need for FP, significantly higher in urban than rural women (22.6% versus 16.6%). Rural women also showed significant lesser odds (adjusted OR: 0.63; 95% CI: 0.461–0.849) of unmet need than the urban counterparts. About 86% of the women had demand for contraception and 77% of them satisfied their demand. The study population revealed a fairly good contraceptive coverage; however, a considerable proportion of women had an unmet need for FP, especially the urban women. The demand for contraception is increasing, and contraceptive services need to expand coverage to marginalized groups in order to reduce the risk of unsafe abortions.

## 1. Introduction

Family planning (FP) is one of the most cost-effective ways of improving the health of women, children, and families [1]. FP and contraception enable young girls, women, and couples to delay, space, and avoid pregnancies [2]. This also prevents high-risk pregnancies, especially in women with high fertility and older age groups [2]. Consequently, FP helps in attaining the desired number of children and a preferable timing of pregnancies, which in-turn prevents unintended pregnancies, and thus, reduces the risk of unsafe abortions [2].

The concept of unmet need for FP points to the gap between the reproductive intentions and the contraceptive behavior of the women [3]. The United Nations (UN) defines the unmet need for FP as: the proportion of women who are fecund and sexually active and want to stop or delay childbearing but are not using any method of contraception [3,4]. According to recent estimations, 214 million reproductive-aged women around the world have an unmet need for modern contraception [2]. The unmet need resulted in unintended pregnancies, as 84% of unintended pregnancies occurred among the women with unmet need [5]. High levels of unmet need for FP and of unintended pregnancies explained the reason for high levels of abortion in countries with legal restriction on abortion [5].

Generally, the extent of unmet need is reduced with an increased use of contraceptives [6]. With understanding the problem of unmet need for FP, the most recent global consensus for FP initiatives came out as a result of the London Summit on FP in 2012. During the summit, stakeholders around the world agreed to combat the problem of unintended pregnancies, which is greatest in the developing regions [7]. The stakeholders agreed to offer support in the provision of contraceptive information, services, and supplies by making them available and affordable in 69 of the worlds’ poorest countries [7], Myanmar included. According to model-based estimates, the global unmet need for FP was reduced from 22% in 1970 to 12% in 2017 by increasing the contraceptive prevalence from 35% to 63% [8].

However, the current norms on increasing preference of smaller families result in increased demand for contraception [8]. Consequently, the total number of women who have an unmet need for FP is increasing despite a global decline in the proportion (percentage) of women with unmet needs. In 2017, it was estimated that ten million more women had unmet needs for FP when compared to the number of women with unmet needs in 2000 [8]. Thus, the problem of unmet need tends to remain constant and increase in some areas. Therefore, the contraceptive prevalence rate (CPR) needs to increase to meet the growing demand [8].

The figures are varied across and within regions as well as countries. In 2015, the contraceptive prevalence in the WHO Southeast Asia region was estimated to be 60% with a 13% unmet need for contraception [9]. According to national surveys in Myanmar, unmet need for FP reduced from 20.6% in 1991 to 19.1% in 1997 and 17.7% in 2007, whereas the CPR increased from 16.8% in 1991 to 41% in 2007 [10]. When the current pregnancy was taken into account, the proportion of unmet need was estimated to be higher as 18.7% in 2007 [10]. These figures revealed only a slight reduction in the extent of unmet need for FP, despite a dramatic increase in CPR.

Consequently, in the costed implementation plan for FP 2020, Myanmar committed to increasing contraceptive prevalence rate (CPR) to over 50% in 2015 and 60% in 2020 through scaling up the provision of integrated birth spacing services [11]. In addition to the provision of FP and contraceptive services by the public sector, the United Nations Population Fund (UNFPA) is providing FP commodities to the public sector as well as other FP implementing partners such as the non-governmental organizations (NGOs) and international non-governmental organizations (INGOs) [11,12]. According to Myanmar Demographic and Health Survey (MDHS) 2015–2016, the targeted CPR for 2015 had already been achieved as 51% of the currently married women were using modern methods of contraceptives [13]. However, only 75% of the potential demand met their FP needs, with 16.2% of married women reporting unmet needs [13]. Thus, the efforts fell short as the commitment was to increase the demand satisfaction to 80% and reduce the FP unmet need to less than 10% in 2015 [11].

The national surveys provide health data that is disaggregated on socio-demographic characteristics of the population, which is useful for identifying the disadvantaged groups across the regional/provincial level [14]. However, there are limitations in the disaggregated data for subnational levels and sub-populations beyond the regional or provincial level [14]. Moreover, rapid urbanization has profound implications on population health [15]. Yangon is the most populated region in Myanmar with rapid urbanization, and 80% of the growth of the Yangon population in the five years preceding the 2014 census was due to internal migration [16,17]. Within the cities, poor social and living conditions, such as those encountered in ghettos and slums, contribute to greater health problems [14]. Thus, disaggregation of health and health-determinant data to reveal the urban health inequities within the cities is also crucial [15].

Thus, this study aimed to: (i) measure the extent of unmet need for family planning among 18–49-year-old married urban and rural women in the Yangon region (south and north), their demand, and satisfaction with family planning (ii) estimate the potential for contraceptive use in the future among the group with unmet needs, and (iii) explore the reasons for not using contraceptives among the group.

## 2. Materials and Methods

**Figure 1:**
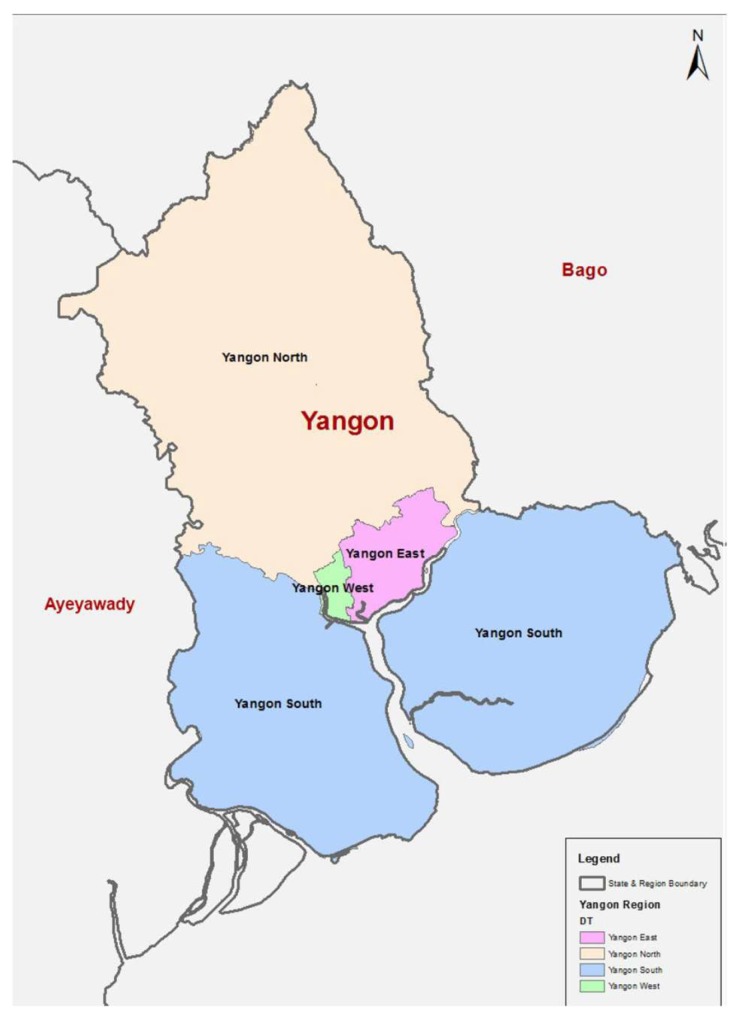
Map of the Yangon Region by Districts. Source: The 2014 Myanmar Population and Housing Census: Yangon Region Report. [17]

A cross-sectional study was conducted in the Yangon region. Yangon is the most populated region in Myanmar, with a population of more than seven million people [17]. The Yangon region is divided into four parts (districts) whereby the east and west districts form central Yangon, which has an almost urbanized population [17]. On the other hand, the south and north districts are situated on the periphery of Yangon with a mixed urban and rural population [17]. The urban areas in the south and north parts are located at the peripheral out-skirts of Yangon city (see Yangon region map in Figure 1), forming the peri-urban slum areas. Moreover, many industrial zones have been established in Yangon, especially in the north, attracting migration from different parts of the country [16]. Since the population in south and north Yangon is a mixture of different social-economic backgrounds, we purposively selected them as our study sample.

A multistage cluster sampling method was used; proportionally weighted to the population size as per the Yangon region census report. The sample size was determined by taking the contraceptive prevalence of 46% as estimated in the Multiple Indicators Cluster Survey 2009–2010 [18]. We randomly selected 8 wards and 8 villages from each district, among 110 wards and 375 villages from the south district, and 125 wards and 235 villages from the north district, totaling to 32 sampling units. The list of wards and villages was based on the 2014 Yangon Region census report.

**Figure 2:**
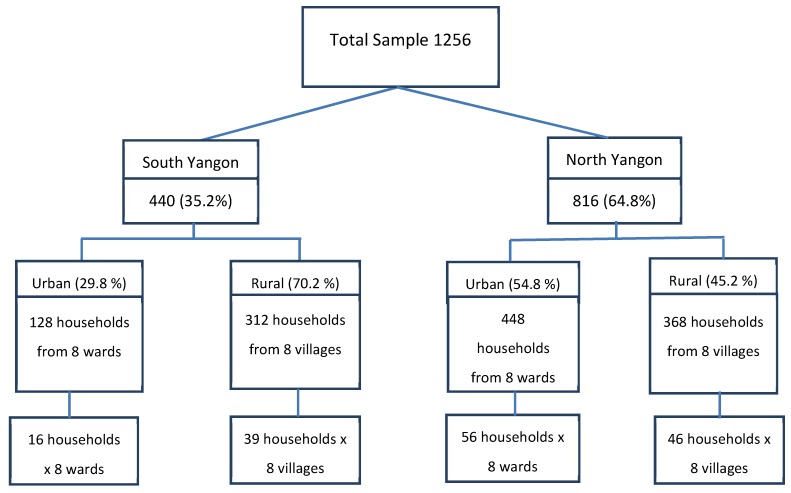
Sampling frame.

Afterward, the households were randomly selected from the household list of the 32 wards and villages. Randomization was done to get 128 and 448 households from urban wards and 312 and 368 households from rural villages of south and north Yangon, respectively. The numbers of households were distributed between urban and rural and between south and north parts according to differences in population size and urban/rural proportions in south and north Yangon. The total number of selected households, and thus, the number of invitees was 1256. With a response rate of 87.6%, the total sample included for analysis was 1100 (see float chart in Figure 2).

After getting approval from the Ethical Committee of Medical Research (IRB number: IRB00008835), Myanmar and NSD (Norsk senter for forskningsdata: Norwegian centre for Research data) and pretesting data collection tools, data collection was conducted by trained interviewers. The participants were interviewed using structured questionnaires with content similar to the reproductive health questions of the demographic and health surveys [19], and some added detailed questions on contraceptives use throughout the studied women’s reproductive life. Invitations and informed consent forms were sent to the selected households after which consenting respondents were recruited. Currently-married women in the reproductive age (18–49 years) were included in this study. Unmarried women, separated/divorced women, and widows were excluded as childbearing and contraceptive uses are generally confined to married couples in Myanmar [10].

Completeness of the data was checked in the field, and data entry was done by Epidata 3.1 (developed by the Epidata Association based in Odense, Denmark). All the data were entered anonymously using an assigned ID number.

### 2.1. Variables

The background characteristics of the respondents were reviewed and described as: urban/rural residence (as per stated in the Yangon region census report), age—grouped into seven groups: (18–19, 20–24, 25–29, 30–34, 35–39, 40–44, 45–49), education level—low (read and write), moderate (primary and middle school level), and high (high school and university level), occupation of the respondent, family size (5 members or less and more than 5 members) and type (nuclear or extended). The number of children (parity) was grouped into zero (no child), women with 1 or 2 children, women with 3 or 4 children, and women having 5 children and above. The household income was grouped according to the percentiles: <25th percentile as low, 25th to 75th percentile as moderate, and >75th percentile as good. Accessibility to the health facility was measured in terms of traveling time and grouped as accessible within 15 min, 15 to 30 min, and more than 30 min to the nearest health facility.

In order to identify women with unmet need, we explored the status of contraception (currently using or not), pregnancy status (currently pregnant or not), the stated desire for the current pregnancy for pregnant women (was it planned or unplanned), and for the last birth (planned or unplanned/mistimed/unintended). We also assessed the fecundity status by seeking information on the last menstrual period, menopausal or not, and whether postpartum amenorrhoeic or whether she could not get pregnant for 5 years in spite of not using contraceptives. In addition, we explored women’s desire for having more children and the timing in which they wanted to have children.

The unmet need for family planning (FP) among the participants was determined by applying the United Nations (UN) definition for unmet need in currently married women or women in-union [4]. The variable—unmet need for FP was constructed by the summation of three groups (Supplemenatry Materials). The first group comprised women who were currently not pregnant and not postpartum amenorrhoeic (those responding that they do not want a child soon or want to stop childbearing) and currently not using contraceptives. The second group comprised women who were currently pregnant with the pregnancy being unplanned (mistimed). The last group comprised women who were postpartum amenorrhoeic who had had an unplanned last birth (within the past 2 years). In this study, the group with unmet needs was derived from fecund women (women who are fertile). Thus, the menopausal women and women who cannot get pregnant were excluded from the unmet need group.

The total demand for FP was determined by the summation of the women who were currently using any contraceptive method and the women having an unmet need for FP [4]. The satisfaction of demand for FP (modern methods) was obtained by dividing the current contraceptive users (modern methods) with the total demand for FP [4].

The stated intention for contraceptive use in the future: that will or will not use was also checked. And the reasons for not using contraceptives among the non-users were also explored to get information for tailoring the approach to attract the non-users.

### 2.2. Statistical Methods

Bivariate differences in socio-demographical background by urban/rural residence were tested using chi-square tests. The CPR and the unmet need for FP among urban and rural women were also tested by chi-square. The proportion of the unmet need group according to the fertility intention (to limit or to space the births) was presented in percentages.

**Figure 3:**
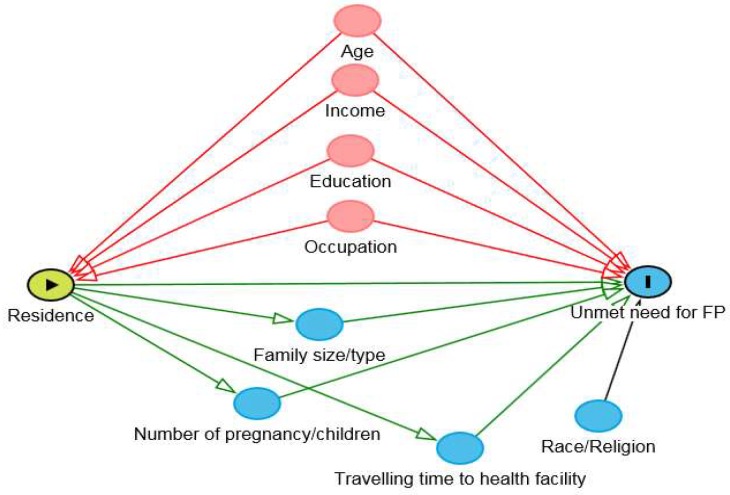
Directed acyclic graph (DAG) on the association between urban-rural residence and unmet need for family planning among 18–49-year-old married women in south and north Yangon.

The association between urban-rural residence and unmet need for FP was estimated in logistical regression (Model 1, crude model). Based on a directed acyclic graph (DAG) [20], as shown in Figure 3, we identified the following confounders: age, education, income, and occupation, and adjusted in the logistic regression analyses. The family size and type, number of pregnancy and children, and traveling time to health facilities were considered as mediators between residence and unmet need, and thus, not adjusted for logistic analysis.

The potential confounders: respondent’s age, education, occupation, and household income were adjusted in Model 2. In addition to the potential confounders adjusted in Model 2, age-square was also adjusted in Model 3. The odd ratios with 95% CI and *p* values for all three models were compared. Interaction between urban/rural living and education level, occupational status, and household income were tested for robustness by plotting delta-beta to access the influence of outliers, but there was no significant outlier.

The satisfaction of demand for FP with modern methods was also calculated and analyzed among urban and rural women. Furthermore, the unmet need group was analyzed according to the past experience of contraception and stated the intention for future use; to estimate the potential of contraceptive use in the future [21].

All the analysis was carried out by Stata software version 15, after checking and cleaning the data set. The ‘svyset’ command was used during the analysis, after declaring the complex survey design with weighting at different stages of sampling according to the Yangon region population data of the 2014 Myanmar Census [17].

## 3. Results

A total of 1100 (505 urban and 595 rural) currently married women participated in the survey. There were few (less than 10) missing observations in the background characteristics (education, income, occupation, family size/type, and traveling time to a health facility). No missing values were observed in age, parity, and responses about fertility and contraceptive use.

The educational level and household income were significantly better in urban than in rural women (Table 1). About half of the women were housewives, both in urban and rural areas, and were busy with daily work (unpaid) of taking care of the baby, family, and the household. The family size and type also significantly differed as a greater proportion of the rural women lived with their own family (nuclear family) and had quite a small family size (five members or less). While a quarter of the urban women lived with extended family with their parents or in-laws/relatives which almost coincided with having a larger family size. The accessibility to the health facility, age distribution, and parity did not significantly differ between urban and rural women.

At the time of the survey, 67.2% of the respondents (739 women) were using some form of contraceptives with a lesser percentage in the urban (63%) than the rural (70%) women (*p* value < 0.05). (not shown in the Table).

After applying the UN definition of unmet need for FP, we found that 213 women, 19.4% (95% CI: 16.7%–22.4%) of the study population had an unmet need for FP. A bivariate analysis of unmet need for FP and urban/rural residence revealed that 22.6% (95% CI: 19.4%–26.1%) of urban women and 16.6% (95% CI: 13.2%–20.8%) of rural women had unmet need (*p* value < 0.05). Within the unmet need group, 132 women (12.0%) had an unmet need for limiting (to stop childbearing), as they did not want more children and 81 (7.4%) had an unmet need for spacing, as they wanted to postpone their births.

The relationship between unmet FP needs and urban/rural residence was analyzed by logistic regression (based on DAG), with consideration of potential confounders. Lesser odds of unmet need for FP were found in rural women, both in the crude (Model 1) and adjusted model (Model 2). No significant interaction was found with the other three potential confounders; this means that education, occupation, and household income did not have any different effect on the urban and rural women. However, the age of respondents showed a significant relationship with the unmet need for FP. Thus, the age-square was added to the adjusted model (Model 2) to get Model 3, and the estimates were presented in Table 2.

The rural women had significantly lesser odds of unmet need for FP when compared to the urban women in all the three models. Model 3, in which the potential confounders (age, education, occupation, household income, and age-square) had been adjusted, was the best fit model as it showed the narrowest confidence interval. Thus, the rural women showed 37% lesser odds of unmet need for FP than their urban counterparts.

The summation of women with unmet need for FP and current users of any type of contraceptive revealed that 943 women (85.7%) in the study population had demand for FP; 426 women from urban areas and 517 women in the rural parts. Among them, 726 women (311 urban and 415 rural) were currently using some form of modern contraceptive. Hence, 77% of the women who had demand for FP were satisfied with modern methods. The demand satisfaction was higher in the rural women when compared to the urban women: 80% versus 73% (*p* = 0.031).

**Figure 4:**
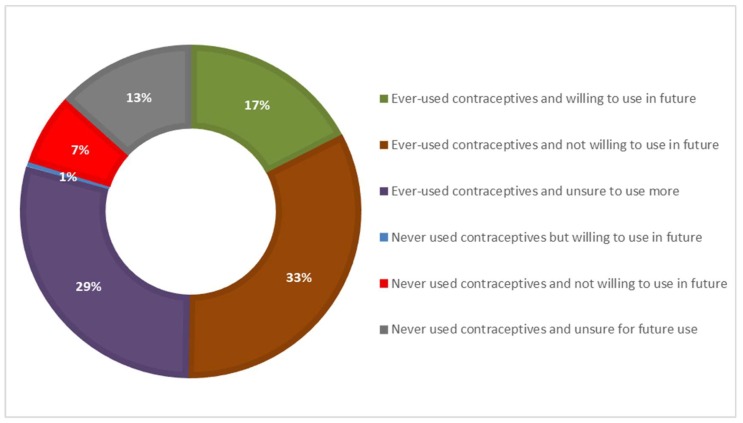
Percentage distribution of previous use and non-use of contraceptives and intention to use in the future; among the women with unmet need for family planning.

In order to estimate the potential for future contraception, the women in the unmet need group were classified according to their past experience of contraceptive use and the desire to use contraceptives in the future (as demonstrated in Figure 4). About 40% of the women having an unmet need for FP were unsure or undecided about future contraception.

A large proportion of the women (nearly 80%) within the unmet need group had ever-used contraceptives. Only 18% of the unmet need group were willing to use contraceptives in the future. The women who had previously used contraceptives but were unsure about future use formed the second largest group among the six categories; they also seemed to have potential for contraception use. However, 70 women stated that they did not want to use contraceptives in the future despite having previously used contraception. These women constitute the largest group among the six categories. Moreover, 7% of the women had never used contraceptives and were also not willing to use it in the future, forming the least likely group to use contraceptives.

Regarding the reasons for contraceptive non-use, women who previously used contraceptives cited the most common reasons for discontinuation as: for purposes of having a child, infrequent sex or the husband being away, and having problems with the method used. The common reasons given by current non-users were: the respondent’s opposition, having infrequent sex, and being afraid of side effects (health concern).

## 4. Discussion

In our study, 67.2% of the respondents were currently using some form of contraceptive. Although the income and education levels were significantly higher in urban women, the rural women reported a greater use of contraceptives (70%) than urban women (63%). However, not all the contraceptive non-users were in need of contraception. Some of the women were not using contraceptives because they wanted to conceive, while others had already reached menopause. Thus, we sought to find out the women in need of contraception and checked whether or not they were using contraceptives. We used the UN definition of unmet need for FP in order to be able to compare our findings with other studies.

The unmet need for FP was 19.4%, with a greater percentage in urban (22.6%) than rural (16.6%) currently married women living in south and north Yangon. The logistic regression also revealed lesser odds of unmet need for FP in rural than urban women even with the adjustment of the potential confounders. Our findings indicated a higher proportion of unmet need for FP among currently married women in the Yangon region when compared to the 2015–2016 MDHS, which reported a lower percentage of unmet need for FP (12%) [13]. Despite our study showing a greater proportion of unmet needs, the CPR (67.2%) was a bit higher than the MDHS result (62.7%). This means that more women are demanding for contraception. Consequently, this survey showed greater demand for contraception (85.7%) than the MDHS result (74.5%); the higher demand contributes to a lower proportion of demand satisfaction (77%) when compared to 81% reported in MDHS [12].

In 2018, a study on FP in a township (Natmauk) in central Myanmar revealed similar results; the CPR was 71.7%, and the unmet need for FP was 18.1% in married women of reproductive age [22]. Despite the fact that married women are increasingly using contraceptives, the extent of unmet need is quite high as there is an increasing demand for contraception.

On the other hand, the difference in CPR and unmet need for FP between our study and the MDHS results can be attributed to the sample selection. The MDHS covers the whole country, and the estimates for the Yangon region include all four parts, while only two districts (south and north) were purposively selected for our study. The east and west Yangon regions are located in the central part of the region and have an affluent population with higher social status [17]. However, the south and north are on the periphery of Yangon and consist of peri-urban slum areas where it is somewhat difficult to access formal healthcare facilities. A consultancy report on “Health care for urban poor in Myanmar” raised the issue of rapid rural-urban migration that has increased the number of poor and slum populations [23]. The report also stated that the urban poor have limited access to the existing health care facilities, despite the availability of free services and commodities [23]. A multimethod study by Grace Sheehy (2014) on dynamics shaping access to reproductive health services in peri-urban Yangon, found extant socio-economic, cultural, and geographical barriers in spite of an overarching available reproductive health system [24]. Thus, migration and urbanization contribute to the greater unmet need for FP among the urban women in the study area, signaling the need for contraceptive services to focus more on these areas.

Among the unmet need group, the percentage was higher in the limiters (12%) than spacers (7.4%). The MDHS also reported greater unmet needs in the limiters (9.5%) among currently married women in the Yangon region when compared to the spacers (2.4%) [13]. This indicates the need for contraceptive services, especially the long-acting and permanent methods. According to the 2017 Myanmar FP landscape analysis, at least three modern contraceptives (oral pills, injectable, male condom, or emergency pills) were available in most (more than 80%) of the observed health facilities [12]. However, services for intra-uterine devices (IUDs) were available at 60% of the health facilities, and only 33% of the health facilities were providing services for implants and permanent methods like sterilization [12].

The greater unmet need in women who do not want any more children is alarming as no desire to have children could increase the risk of unsafe abortions, considering the fact that abortion is under legal restriction in Myanmar [25,26]. On checking the hospital statistics for 2016, 54,305 abortion cases were noted in Myanmar, and the abortion rate (as a percentage of total deliveries and abortion cases) was 12.2. A total of 11,779 abortions were reported in the Yangon region with an abortion rate of 13.7—a higher abortion rate than the national average [27]. Moreover, the Myanmar hospital statistics report indicated that unspecified abortions were the number one leading cause of maternal mortality in 2016 [27]. The hospital statistics did not specify the causes and types of abortions.

Although the cause and type of abortion were unspecified, the Yangon region holds quite a large number of abortions and the problem of unmet need could increase the abortion rate. Most of the abortion-related health problems arise from unsafe abortions [25]. It is estimated that if all the women in need of contraception have met needs, the induced abortions occurring in the developing world would reduce by 67%, and the number of maternal deaths as a consequence of unsafe abortion would decline from 22,000 to 4200 [25]. Despite abortion being legal in India, 89,447 abortion cases were reported in 2007–2011; 67% of these were unsafe abortions and 253 abortion-related deaths occurred [28].

In addition to the extent of unmet need, the analysis of future intention to use contraceptives among the unmet need group will be useful for tailoring the approaches to attract the uncovered group. Women who have ever-used contraceptives are more likely to use contraceptives in the future when compared to those that have never used them. However, a large proportion of women in the unmet need group who had ever-used contraceptives did not intend to use them in the future. This indicates that the existence of barriers outweighs their desire to space or limit childbearing; there is a need to explore the factors hindering contraceptive use [29]. More efforts are needed to cover this group as they already have knowledge and experience of contraceptive use but are unwilling to continue due to negative experiences [29]. There could be some constraints in method accessibility and availability as some women asked about implants because they did not know where to get them. And some women have problems with cost as they have to obtain the method from the private sector and it’s difficult to gain access to the public sector during the opening hours because of time constraints. More qualitative research is needed to explore the barriers/hindering factors in more detail in order to provide precise information to program implementers.

The most common reasons for contraceptive non-use among the studied women were problems with the methods used and fear of side effects. Contraceptive counseling on side effects, including method switching when experiencing side effects, should be prioritized with alternative methods being made available [6,30]. The respondents’ opposition to contraception also indicates the need for reproductive health education on the importance of family planning and contraception.

This study has provided comparable results to other surveys by using similar questions to the DHS and UN’s definition of unmet need for FP. However, the study is not without limitations. The limitations of the study are: the study only included currently married women living in two districts of the Yangon region. Even though a large proportion of women at a reproductive age remain unmarried, childbearing mostly takes place within marriage in Myanmar [10]. As such, we did not include unmarried women in this study. This creates a bias and reduces the number of women eligible for the denominator somewhat inflating the proportions. In Myanmar, it is socially unacceptable to ask fertility questions to unmarried women [31,32]. For that reason, questions on non-marital fertility have not been included in the population censuses or nationally representative surveys [31,32]. Therefore, the study findings do not represent all women and the Yangon region as a whole.

## 5. Conclusions

This study points out the need for family planning services among married women in south and north Yangon, and more importantly, the urban women living in the area. Urbanization and increasing demand for contraception in the population imply the greater unmet need for FP. The service providers and program implementers should not only be satisfied with an increasing contraceptive prevalence but also realize that an increase in demand calls for more service expansion. An expansion of contraceptive services will reduce unplanned or unintended pregnancies, and the risk of unsafe abortions, which lead to poor maternal and child health outcomes. Furthermore, there is a need for exploration of contraceptive use among the unmarried women and barriers/hindering factors for contraception with an in-depth discussion, especially among non-users.

## Figures and Tables

**Table 1:** Socio-demographic indicators by urban-rural residence among 18–49-year-old currently married women in south and north Yangon.

Socio-Demographic Characteristics	Urban (%)	Rural (%)	Total (%)	*p* Value
Education				0.002
Low	16 (3.1%)	37 (6.3%)	53 (4.8%)	
Moderate	276 (55.4%)	428 (72.6%)	704 (64.6%)	
High	207 (41.5%)	126 (21.1%)	333 (30.6%)	
Household Income				0.008
Low	109 (21.7%)	186 (31.7%)	295 (27.1%)	
Moderate	236 (47.2%)	291 (49.0%)	527 (48.1%)	
Good	157 (31.1%)	115 (19.3%)	272 (24.8%)	
Occupation				
Civil or Private servant	29 (5.8%)	28 (4.8%)	57 (5.3%)	
Farmer	3 (0.6%)	55 (9.4%)	58 (5.3%)	
Housewife	274 (54.4%)	286 (48.5%)	560 (51.3%)	
Vendor	95 (18.7%)	100 (16.9%)	195 (17.8%)	
Unstable job/daily wages	58 (11.8%)	76 (13.0%)	134 (12.4%)	
Own business	44 (8.7%)	44 (7.4%)	88 (8.0%)	
Traveling time to Health facility				0.193
within 15 min	376 (74.8%)	396 (67.1%)	772 (70.7%)	
15 to 30 min	113 (22.4%)	158 (26.5%)	271 (24.6%)	
>30 min	14 (2.7%)	38 (6.4%)	52 (4.7%)	
Family type				0.015
Nuclear	375 (74.6%)	492 (82.8%)	867 (79.0%)	
Extended	129 (25.4%)	102 (17.2%)	231 (21.0%)	
Family size				0.0001
Having 5 members or less	371 (73.8%)	501 (84.4%)	872 (79.5%)	
More than 5 members	132 (26.2%)	93 (15.6%)	225 (20.5%)	
Age group				0.592
18–19 years	14 (2.7%)	9 (1.5%)	23 (2.1%)	
20–24 years	46 (9.2%)	60 (10.1%)	106 (9.7%)	
25–29 years	91 (18.1%)	106 (17.8%)	197 (18.0%)	
30–34 years	93 (18.4%)	115 (19.4%)	208 (18.9%)	
35–39 years	115 (22.9%)	122 (20.5%)	237 (21.6%)	
40–44 years	90 (17.7%)	101 (16.9%)	191 (17.3%)	
45–49 years	56 (11.0%)	82 (13.8%)	138 (12.5%)	
Parity				0.168
0	68 (13.5%)	57 (9.7%)	125 (11.5%)	
1–2	281 (55.6%)	337 (56.5%)	618 (56.1%)	
3–4	119 (23.5%)	162 (27.3%)	281 (25.5%)	
5 & above	37 (7.4%)	39 (6.5%)	76 (6.9%)	

**Table 2:** The association between urban/rural residence and unmet need for family planning (FP) among 18–49-year-old married women in south and north Yangon**.**

	Model 1 (Crude)	Model 2 (Adjusted) *	Model 3 (Adjusted) **
Odds Ratio ^#^	0.68	0.64	0.63
95% CI	0.496–0.938	0.468–0.882	0.461–0.849
P value	0.020	0.008	0.004

* Model 2, adjusted for age, education, occupation, and household income; ** Model 3, adjusted for age, education, occupation, household income, and age-squared; ^#^ Urban residence is the reference.

## Supplementary Materials

The following are available online at https://www.mdpi.com/1660-4601/16/19/3742/s1, the revised definition of Unmet Need for Family Planning that used for constructing the variable. Unmet need for Family Planning is available online at https://dhsprogram.com/topics/Unmet-Need.cfm.
