# Supplementary material for: Unmet Need for Family Planning among Urban and Rural Married Women in Yangon Region, Myanmar—a Cross-Sectional Study
Source: Int J Environ Res Public Health. 2019 Oct 4;16(19):3742. doi: 10.3390/ijerph16193742 (PMC6801744; doi:10.3390/ijerph16193742)
Supplement: Supplementary file 1 [file ijerph-16-03742-s001.zip › supplementary/DMR approval Page 1.pdf]

The Government of the Republic of the Union of Myanmar
Ministry of Health and Sports
Department of Medical Research
No. 5, Ziwaka Road, Dagon Township, Yangon 11191
Tel : 95-1-375447, 95-1-375457, 95-1-375459 Fax : 95-1-251514

ERC Number: 010416
Approval Number: Ethics/DMR/2016/117
Date of Approval: 27 September, 2016 (valid up to 26 September, 2017)

Project Title: **Trends and Dynamics of Family Planning, Antenatal care, Delivery by skilled birth attendants and Fertility situation in Yangon Region, Myanmar**

Principal Investigator: Dr. Myint Myint Wai
Department of Medical Services

Documents Accepted:

1. Ethical Proposal Form version 2.0 Dated 9 September, 2016
2. Full Proposal Protocol version 2.0 Dated 9 September, 2016
3. Proposal Summary version 2.0 Dated 9 September, 2016
4. Agreement to comply with ethical guideline version 2.0 Dated 9 September, 2016
5. Informed Consent Form (English & Myanmar) version 2.0 Dated 9 September, 2016
6. Questionnaires (English & Myanmar) version 2.0 Dated 9 September, 2016
7. Investigators' CV version 2.0 Dated 9 September, 2016

The Ethics Review Committee on Medical Research Involving Human Subjects, Department of Medical Research, Ministry of Health and Sports approves to conduct the proposed research project as it is in full compliance with the Declaration of Helsinki, Council for International Organizations of Medical Sciences guidelines and International Conference on Harmonisation in Good Clinical Practice guidelines.

Prof. Pe Thet Khin
Chairperson
Ethics Review Committee
Department of Medical Research
