# Supplementary material for: Unmet Need for Family Planning among Urban and Rural Married Women in Yangon Region, Myanmar—a Cross-Sectional Study
Source: Int J Environ Res Public Health. 2019 Oct 4;16(19):3742. doi: 10.3390/ijerph16193742 (PMC6801744; doi:10.3390/ijerph16193742)
Supplement: Supplementary file 1 [file ijerph-16-03742-s001.zip › supplementary/DMR approval Page 2.pdf]

The Government of the Republic of the Union of Myanmar
Ministry of Health and Sports
Department of Medical Research
No. 5, Ziwaka Road, Dagon Township, Yangon 11191
Tel : 95-1-375447, 95-1-375457, 95-1-375459 Fax : 95-1-251514

Approval is subject to following conditions:

- The principal investigator (PI) must notify immediately to the ERC of any changes or deviation in the conduct of the research activity. Only with the ERC's approval such changes in the study must be pursued. The PI must also make a prompt report to the ERC of any new and significant information that may impact a research subject's safety or willingness to continue in the study and any anticipated problems involving risks to the participants or other.

- PI is responsible for submitting the progress report at least 6 weeks prior to the expiry of the approved date to allow adequate time for the ERC for substantive and meaningful review and for assuring that the research is not conducted beyond the approved date.

- Final report is to be provided to ERC at the end of the study.

- Random site visits may be carried out to ensure that informed consent procedures are appropriate.
