# Supplementary material for: Unmet Need for Family Planning among Urban and Rural Married Women in Yangon Region, Myanmar—a Cross-Sectional Study
Source: Int J Environ Res Public Health. 2019 Oct 4;16(19):3742. doi: 10.3390/ijerph16193742 (PMC6801744; doi:10.3390/ijerph16193742)
Supplement: Supplementary file 1 [file ijerph-16-03742-s001.zip › supplementary/NSD meldeskjema sendt 23September2016.pdf]

MELDESKJEMA

Meldeskjema (versjon 1.4) for forsknings- og studentprosjekt som medfører meldeplikt eller konsesjonsplikt
(jf. personopplysningsloven og helseregisterloven med forskrifter).

1. Intro

Samles det inn direkte personidentifiserende opplysninger?	Ja ● Nei ○	En person vil være direkte identifiserbar via navn, personnummer, eller andre personentydige kjennetegn.<br>Les mer om hva personopplysninger.<br>NB! Selv om opplysningene skal anonymiseres i oppgave/rapport, må det krysses av dersom det skal innhentes/registreres personidentifiserende opplysninger i forbindelse med prosjektet.
Hvis ja, hvilke?	■ Navn<br>□ 11-sifret fødselsnummer<br>■ Adresse<br>□ E-post<br>□ Telefonnummer<br>□ Annet	
Annet, spesifiser hvilke		
Skal direkte personidentifiserende opplysninger kobles til datamaterialet (koblingsnøkkel)?	Ja ● Nei ○	Merk at meldeplikten utløses selv om du ikke får tilgang til koblingsnøkkel, slik fremgangsmåten ofte er når man benytter en databehandler
Samles det inn bakgrunnsopplysninger som kan identifisere enkeltpersoner (indirekte personidentifiserende opplysninger)?	Ja ○ Nei ●	En person vil være indirekte identifiserbar dersom det er mulig å identifisere vedkommende gjennom bakgrunnsopplysninger som for eksempel bostedskommune eller arbeidsplass/skole kombinert med opplysninger som alder, kjønn, yrke, diagnose, etc.<br>NB! For at stemme skal regnes som personidentifiserende, må denne bli registrert i kombinasjon med andre opplysninger, slik at personer kan gjenkjennes.
Hvis ja, hvilke		
Skal det registreres personopplysninger (direkte/indirekte/via IP-/epost adresse, etc) ved hjelp av nettbaserte spørreskjema?	Ja ○ Nei ●	Les mer om nettbaserte spørreskjema.
Blir det registrert personopplysninger på digitale bilde- eller videoopptak?	Ja ○ Nei ●	Bilde/videoopptak av ansikter vil regnes som personidentifiserende.
Søkes det vurdering fra REK om hvorvidt prosjektet er omfattet av helseforskningsloven?	Ja ● Nei ○	NB! Dersom REK (Regional Komité for medisinsk og helsefaglig forskningsetikk) har vurdert prosjektet som helseforskning, er det ikke nødvendig å sende inn meldeskjema til personvernombudet (NB! Gjelder ikke prosjekter som skal benytte data fra pseudonyme helseregistre).<br>Dersom tilbakemelding fra REK ikke foreligger, anbefaler vi at du avventer videre utfylling til svar fra REK foreligger.

2. Prosjekttittel

Prosjekttittel	Vold, mental helse og mødrehelse i Yangon, Myanmar	Oppgi prosjektets tittel. NB! Dette kan ikke være «Masteroppgave» eller liknende, navnet må beskrive prosjektets innhold.

3. Behandlingsansvarlig institusjon

Institusjon	Universitetet i Oslo	Velg den institusjonen du er tilknyttet. Alle nivå må oppgis. Ved studentprosjekt er det studentens tilknytning som er avgjørende. Dersom institusjonen ikke finnes på listen, har den ikke avtale med NSD som personvernombud. Vennligst ta kontakt med institusjonen.
Avdeling/Fakultet	Det medisinske fakultet	
Institutt	Institutt for helse og samfunn	

4. Daglig ansvarlig (forsker, veileder, stipendiat)

Fornavn	Espen	Før opp navnet på den som har det daglige ansvaret for prosjektet. Veileder er vanligvis daglig ansvarlig ved studentprosjekt.<br><br>Veileder og student må være tilknyttet samme institusjon. Dersom studenten har ekstern veileder, kanbiveileder eller fagansvarlig ved studiestedet stå som daglig ansvarlig.<br><br>Arbeidssted må være tilknyttet behandlingsansvarlig institusjon, f.eks. underavdeling, institutt etc.<br><br>NB! Det er viktig at du oppgir en e-postadresse som brukes aktivt. Vennligst gi oss beskjed dersom den endres.
Etternavn	Bjertness	
Stilling	Professor	
Telefon	92412026	
Mobil		
E-post	espen.bjertness@medisin.uio.no	
Alternativ e-post	espen.bjertness@medisin.uio.no	
Arbeidssted	Universitetet i Oslo, Institutt for helse og samfunn	
Adresse (arb.)	Box 1130 Blindern	
Postnr./sted (arb.sted)	0318 Oslo	

5. Student (master, bachelor)

Studentprosjekt	Ja ○ Nei ●	Dersom det er flere studenter som samarbeider om et prosjekt, skal det velges en kontaktperson som føres opp her. Øvrige studenter kan føres opp under pkt 10.

6. Formålet med prosjektet

Formål	Cross-sectional design; Aims:<br>(1) prevalence estimates and associated factors of mental health problems (depression, anxiety, post-traumatic stress disorder) and domestic violence;<br>(2) family planning practices over the life course, including unmet needs and barriers in using family planning and birth spacing practices;<br>(3) to assess socioeconomic equity on utilization of ANC and delivery care by perspectives of women and providers in Myanmar.<br>Will be basis for two PhD candidates on maternal health and one candidate for Mental Health and domestic Violence. Mixed model for studying equity (3): with additional samples of all 157 health providers in study area and in-depth interview of 24 providers and 40 women from the sample.	Redegjør kort for prosjektets formål, problemstilling, forskningsspørsmål e.l.

7. Hvilke personer skal det innhentes personopplysninger om (utvalg)?

Kryss av for utvalg	□ Barnehagebarn<br>□ Skoleelever<br>□ Pasienter<br>□ Brukere/klienter/kunder<br>□ Ansatte<br>□ Barnevernsbarn<br>□ Lærere<br>■ Helsepersonell<br>□ Asylsøkere<br>■ Andre	
Beskriv utvalg/deltakere	The study will include 2400 male and female aged 18-49 years living in urban and rural areas. Estimated response rate 80%. Married women, unmarried women and males will receive different questionnaires.<br>Mixed model for studying equity with additional samples of all 157 health providers in study area and in-depth interview of 24 providers and 40 women from the sample.	Med utvalg menes dem som deltar i undersøkelsen eller dem det innhentes opplysninger om.
Rekruttering/trekking	1. Random sample of 2400 18-49 year olds at household Level. Random villages/urban wards; Random households; random individual from each household;<br>2. all 157 health providers in study area will be included, and in-depth interview of 24 selected providers;<br>3. 40 women among the 2400 participants will be selected for indepth interview. 20 for high and low quintile of household wealth in each of urban and rural area.	Beskriv hvordan utvalget trekkes eller rekrutteres og oppgi hvem som foretar den. Et utvalg kan trekkes fra registre som f.eks. Folkeregisteret, SSB-registre, pasientregistre, eller det kan rekrutteres gjennom f.eks. en bedrift, skole, idrettsmiljø eller eget nettverk.

Førstegangskontakt	1. Village leaders (rural area) and Ward leaders (urban area) will be contacted. The leaders provide a list of households. Random selection of household. PhD-students and Field-workers will contact the selected households and randomly invite one person.<br>2. All Health providers in the area will be invited and approached by the PhD-students and Field-workers.	Beskriv hvordan kontakt med utvalget blir opprettet og av hvem.<br><br>Les mer om dette på temasidene.
Alder på utvalget	□ Barn (0-15 år)<br>□ Ungdom (16-17 år)<br>■ Voksne (over 18 år)	Les om forskning som involverer barn på våre nettsider.
Omtrentlig antall personer som inngår i utvalget	2400	
Samles det inn sensitive personopplysninger?	Ja ● Nei ○	Les mer om sensitive opplysninger.
Hvis ja, hvilke?	□ Rasemessig eller etnisk bakgrunn, eller politisk, filosofisk eller religiøs oppfatning<br>□ At en person har vært mistenkt, siktet, tiltalt eller dømt for en straffbar handling<br>■ Helseforhold<br>■ Seksuelle forhold<br>□ Medlemskap i fagforeninger	
Inkluderes det myndige personer med redusert eller manglende samtykkekompetanse?	Ja ○ Nei ●	Les mer om pasienter, brukere og personer med redusert eller manglende samtykkekompetanse.
Samles det inn personopplysninger om personer som selv ikke deltar (tredjepersoner)?	Ja ○ Nei ●	Med opplysninger om tredjeperson menes opplysninger som kan spores tilbake til personer som ikke inngår i utvalget. Eksempler på tredjeperson er kollega, elev, klient, familiemedlem.

8. Metode for innsamling av personopplysninger

Kryss av for hvilke datainnsamlingsmetoder og datakilder som vil benyttes	■ Papirbasert spørreskjema<br>□ Elektronisk spørreskjema<br>■ Personlig intervju<br>□ Gruppeintervju<br>□ Observasjon<br>□ Deltakende observasjon<br>□ Blogg/sosiale medier/internett<br>□ Psykologiske/pedagogiske tester<br>□ Medisinske undersøkelser/tester<br>□ Journaldata	Personopplysninger kan innhentes direkte fra den registrerte f.eks. gjennom spørreskjema,intervju, tester, og/eller ulike journaler (f.eks. elevmapper, NAV, PPT, sykehus) og/eller registre (f.eks.Statistisk sentralbyrå, sentrale helseregistre).<br><br>NB! Dersom personopplysninger innhentes fra forskjellige personer (utvalg) og med forskjellige metoder, må dette spesifiseres i kommentar-boksen. Husk også å legge ved relevante vedlegg til alle utvalgs-gruppene og metodene som skal benyttes.<br><br>Les mer om registerstudier her.<br><br>Dersom du skal anvende registerdata, må variabelliste lastes opp under pkt. 15
	□ Registerdata	
	□ Annen innsamlingsmetode	
Tilleggsopplysninger		

9. Informasjon og samtykke

Oppgi hvordan utvalget/deltakerne informeres	■ Skriftlig<br>■ Muntlig<br>□ Informeres ikke	Dersom utvalget ikke skal informeres om behandlingen av personopplysninger må det begrunnes.<br><br>Les mer her.<br><br>Vennligst send inn mal for skriftlig eller muntlig informasjon til deltakerne sammen med meldeskjema.<br><br>Last ned en veiledende mal her.<br><br>NB! Vedlegg lastes opp til sist i meldeskjemaet, se punkt 15 Vedlegg.
Samtykker utvalget til deltakelse?	● Ja<br>○ Nei<br>○ Flere utvalg, ikke samtykke fra alle	For at et samtykke til deltakelse i forskning skal være gyldig, må det være frivillig, uttrykkelig og informert.<br><br>Samtykke kan gis skriftlig, muntlig eller gjennom en aktiv handling. For eksempel vil et besvart spørreskjema være å regne som et aktivt samtykke.<br><br>Dersom det ikke skal innhentes samtykke, må det begrunnes.

10. Informasjonssikkerhet

Hvordan oppbevares navnelisten/ koblingsnøkkelen og hvem har tilgang til den?	PhD-student Aye Nyein Moe Myint er den eneste som har tilgang til koblingsnøkkel. Hun trenger nøkkelen for å finne frem til 40 kvinner som skal dybdeintervjues etter den ordinære datainnsamlingen. Koblingsnøkkelen lagres på minnepinne som ligger nedlåst.	
Oppbevares direkte personidentifiserbare opplysninger på andre måter?	Ja ○ Nei ●	
Spesifiser	Papirspørreskjema nummereres. I et eget dokument knyttes nummeret sammen med navn og adresse for å gjøre dybdeintervju av 40 gifte kvinner som har født barn i løpet av siste år.	NB! Som hovedregel bør ikke direkte personidentifiserende opplysninger registreres sammen med det øvrige datamaterialet.
Hvordan registreres og oppbevares personopplysningene?	□ På server i virksomhetens nettverk<br>□ Fysisk isolert PC tilhørende virksomheten (dvs. ingen tilknytning til andre datamaskiner eller nettverk, interne eller eksterne)<br>□ Datamaskin i nettverkssystem tilknyttet Internett tilhørende virksomheten<br>□ Privat datamaskin<br>□ Videoopptak/fotografi<br>□ Lydopptak<br>□ Notater/papir<br>■ Mobile lagringsenheter (bærbar datamaskin, minnepenn, minnekort, cd, ekstern harddisk, mobiltelefon)<br>□ Annen registreringsmetode	Merk av for hvilke hjelpemidler som benyttes for registrering og analyse av opplysninger.<br><br>Sett flere kryss dersom opplysningene registreres på flere måter.<br><br>Med «virksomhet» menes her behandlingsansvarlig institusjon.<br><br>NB! Som hovedregel bør data som inneholder personopplysninger lagres på behandlingsansvarlig sin forskningsserver.<br><br>Lagring på andre medier - som privat pc, mobiltelefon, minnepinne, server på annet arbeidssted - er mindre sikkert, og må derfor begrunnes. Slik lagring må avklares med behandlingsansvarlig institusjon, og personopplysningene bør krypteres.
Annen registreringsmetode beskriv		
Hvordan er datamaterialet beskyttet mot at uvedkommende får innsyn?	Lagres på passordbeskyttet PC	Er f.eks. datamaskintilgangen beskyttet med brukernavn og passord, står datamaskinen i et låsbart rom, og hvordan sikres bærbare enheter, utskrifter og opptak?
Samles opplysningene inn/behandles av en databehandler?	Ja ○ Nei ●	Dersom det benyttes eksterne til helt eller delvis å behandle personopplysninger, f.eks. Questback, transkriberingsassistent eller tolk, er dette å betrakte som en databehandler. Slike oppdrag må kontraktsreguleres.
Hvis ja, hvilken		
Overføres personopplysninger ved hjelp av e-post/Internett?	Ja ○ Nei ●	F.eks. ved overføring av data til samarbeidspartner, databehandler mm.<br><br>Dersom personopplysninger skal sendes via internett, bør de krypteres tilstrekkelig.<br><br>Vi anbefaler for ikke lagring av personopplysninger på nettskytjenester.<br><br>Dersom nettskytjeneste benyttes, skal det inngås skriftlig databehandleravtale med leverandøren av tjenesten.
Hvis ja, beskriv?		
Skal andre personer enn daglig ansvarlig/student ha tilgang til datamaterialet med personopplysninger?	Ja ○ Nei ●	
Hvis ja, hvem (oppgi navn og arbeidssted)?		
Utleveres/deles personopplysninger med andre institusjoner eller land?	● Nei<br>○ Andre institusjoner<br>○ Institusjoner i andre land	F.eks. ved nasjonale samarbeidsprosjekter der personopplysninger utveksles eller ved internasjonale samarbeidsprosjekter der personopplysninger utveksles.

11. Vurdering/godkjenning fra andre instanser

Søkes det om dispensasjon fra taushetsplikten for å få tilgang til data?	Ja ○ Nei ●	For å få tilgang til taushetsbelagte opplysninger fra f.eks. NAV, PPT, sykehus, må det søkes om dispensasjon fra taushetsplikten. Dispensasjon søkes vanligvis fra aktuelt departement.
Hvis ja, hvilke		
Søkes det godkjenning fra andre instanser?	Ja ● Nei ○	F.eks. søke registereier om tilgang til data, en ledelse om tilgang til forskning i virksomhet, skole.
Hvis ja, hvilken	REK i Norge; Etikk komité ved Ministry of Health, Myanmar	

12. Periode for behandling av personopplysninger

Prosjektstart<br><br>Planlagt dato for prosjektslutt	10.10.2016<br><br>10.01.2017	Prosjektstart Vennligst oppgi tidspunktet for når kontakt med utvalget skal gjøres/datainnsamlingen starter.<br><br>Prosjektslutt: Vennligst oppgi tidspunktet for når datamaterialet enten skalanonymiseres/slettes, eller arkiveres i påvente av oppfølgingsstudier eller annet.

Skal personopplysninger publiseres (direkte eller indirekte)?	□ Ja, direkte (navn e.l.)<br>□ Ja, indirekte (bakgrunnsopplysninger)<br>■ Nei, publiseres anonymt	NB! Dersom personopplysninger skal publiseres, må det vanligvis innhentes eksplisitt samtykke til dette fra den<br>enkelte, og deltakere bør gis anledning til å lese gjennom og godkjenne sitater.
Hva skal skje med datamaterialet ved prosjektslutt?	■ Datamaterialet anonymiseres<br>□ Datamaterialet oppbevares med personidentifikasjon	NB! Her menes datamaterialet, ikke publikasjon. Selv om data publiseres med personidentifikasjon skal som regel øvrig data anonymiseres.Med anonymisering menes at datamaterialet bearbeides slik at det ikke lenger er mulig å føre opplysningene tilbake til enkeltpersoner.<br><br>Les mer om anonymisering.
**13. Finansiering**		
Hvordan finansieres prosjektet?	Norad. NORHED-Project: MMY-13/0049 “Health and Sustainable Development in Myanmar – Competence Building in Public Health and Medical Research and Education (MY-NORTH)	
**14. Tilleggsopplysninger**		
Tilleggsopplysninger		
